# Evidence of SARS-CoV-2 Exposure in Rodents from Rural Localities in the Yucatan Peninsula, Mexico

**DOI:** 10.3390/v18040435

**Published:** 2026-04-03

**Authors:** Ana Laura Vigueras-Galván, Montserrat Elemi García-Hernández, Karen Cortés-Sarabia, Oscar Del Moral-Hernández, Sofía L. Alcaraz-Estrada, Benjamín Roche, Audrey Arnal, Gerardo Suzán, Rosa Elena Sarmiento-Silva

**Affiliations:** 1 Laboratorio de Virología, Departamento de Microbiología e Inmunología, Facultad de Medicina Veterinaria y Zootecnia, Universidad Nacional Autónoma de México, Av. Universidad #3000, Col. UNAM-CU, Coyoacán, Ciudad de México ZP 04510, Mexico; 2 Laboratorio de Inmunobiología y Diagnóstico Molecular, Facultad de Ciencias Químico-Biológicas, Universidad Autónoma de Guerrero, Calle Lázaro Cárdenas, Col. El Centenario, Chilpancingo de Bravo ZP 39086, Guerrero, Mexico; 3 Laboratorio de Virología, Facultad de Ciencias Químico-Biológicas, Universidad Autónoma de Guerrero, Calle Lázaro Cárdenas, Col. El Centenario, Chilpancingo de Bravo ZP 39086, Guerrero, Mexico; 4 División de Medicina Genómica, Centro Médico Nacional 20 de Noviembre-ISSSTE, Av. Félix Cuevas #540, Col. del Valle Sur, Benito Juárez ZP 03104, Mexico; 5 Maladies Infectieuses et Vecteurs: Écologie, Génétique, Évolution et Contrôle, Université de Montpellier, Institut de Recherche pour le Développement, Centre National de la Recherche Scientifique, 911 Avenue Agropolis, BP 64 501, ZP 34394 Montpellier, France; 6 Departamento de Etología, Fauna Silvestre y Animales de Laboratorio, Facultad de Medicina Veterinaria y Zootecnia, Universidad Nacional Autónoma de Mexico, Av. Universidad #3000, Col. UNAM-CU, Coyoacán, Ciudad de México ZP 04510, Mexico

**Keywords:** reverse zoonosis, SARS-CoV-2, rodents, rural communities, Yucatan Peninsula

## Abstract

Zoonotic diseases involve pathogen transmission between humans and animals, with most research focused on animal-to-human spillover. However, reverse zoonosis—the transmission of pathogens from humans to animals—remains understudied despite its potential ecological and epidemiological consequences. The SARS-CoV-2 pandemic highlights this risk, as human-associated viruses may sporadically infect wildlife species and generate novel exposure pathways. To assess evidence of SARS-CoV-2 exposure in wildlife, we analyzed serum and rectal swab samples from rodents collected in rural localities of the Yucatan Peninsula, Mexico, between 2021 and 2022. An indirect ELISA detected antibodies against SARS-CoV-2 in 23.1% of sampled rodents. Molecular analysis detected one positive sample with a pan-coronavirus RT-PCR, though all were negative for SARS-CoV-2–specific assays. This study provides serological evidence of SARS-CoV-2 exposure in rodent communities from rural areas of Mexico and is consistent with sporadic wildlife spillback events rather than sustained transmission. The observed exposure patterns may be influenced by human activities and frequent human–wildlife interactions in heterogeneous rural landscapes. Our results underscore the need for integrated serological and genomic surveillance to better understand the ecological context of reverse zoonosis and its implications for public health.

## 1. Introduction

Severe Acute Respiratory Syndrome Coronavirus 2 (SARS-CoV-2), formally classified as *Betacoronavirus pandemicum* within the genus *Sarbecovirus*, is the etiological agent of Coronavirus Disease 2019 (COVID-19) and has caused an unprecedented global pandemic since its emergence in late 2019. As of 2024 COVID-19 has resulted in over 7 million deaths worldwide and substantial economic losses [1]. Beyond its immediate health and economic impacts, the pandemic has highlighted the complex interactions between humans, animals, and ecosystems, emphasizing the need to better understand the mechanisms underlying zoonotic and reverse zoonotic transmission events. SARS-CoV-2’s ability to infect a broad range of mammalian hosts raises concerns about its potential establishment in wildlife reservoirs, which could complicate long-term surveillance and control efforts and pose additional challenges for public health.

After the emergence of SARS-CoV-2, human-to-animal transmission was documented in a wide range of vertebrate species, including pets (cats, hamsters, ferrets and dogs), captive wild felids, mink, white-tailed deer and non-human primates [2,3]. Bidirectional transmission has been conclusively demonstrated only in limited contexts, most notably in mink farms, where human-to-animal and subsequent animal-to-human transmission were confirmed through whole-genome sequencing [4]. During the SARS-CoV-2 pandemic, wildlife rehabilitation centers were affected in multiple ways, including an increase in the release of animals due to reduced economic resources. These circumstances may have increased the risk of viral dissemination in natural environments, either through environmental contamination (e.g., water or fomites) or through the release of apparently asymptomatic infected animals, posing a potential threat to particularly susceptible wildlife taxa such as felids, mustelids, and non-human primates [5]. Systematic reviews adopting a One Health perspective have highlighted that, despite extensive reports of SARS-CoV-2 infection in domestic animals and captive wildlife, evidence from free-ranging wildlife remains scarce and uneven across taxa and regions [6].

Rodents are one of the most diverse groups of vertebrates in the world, with over 2000 species, representing more than 40% of all mammalian species. They are widely distributed in different types of habitats and are recognized as a major zoonotic source of human infectious diseases [7]. Synanthropic rodent species are tolerant to disturbances and can adapt to anthropogenic habitat modifications. This allows them to act as bridges for interspecific viral transmission by participating in spillover events.

Evidence from evolutionary and experimental studies suggests that SARS-CoV-2 has the capacity to adapt to rodent hosts, including the acquisition of mutations in the Spike protein gene that enhance infectivity in this group, some of which resemble mutations identified in the Omicron variant. It has been hypothesized that the ancestral lineage of Omicron may have involved a human-to-rodent transmission event, during which the virus accumulated adaptive mutations before spilling back into the human population [8]. In parallel, multiple rodent species have been evaluated for their potential to act as SARS-CoV-2 reservoirs or as experimental models for respiratory infection, primarily under controlled laboratory conditions [9,10,11].

There are, however, few studies that have investigated SARS-CoV-2 infection in rodents within wildlife [12]. Recent studies from Southeast Asia have reported serological evidence of SARS-CoV-2 exposure in several rodent species, highlighting the potential role of rodents in reverse zoonotic transmission and underscoring the need for regional studies in other biodiverse regions [13]. In contrast, surveillance efforts in North America and Europe have yielded extremely limited and often inconclusive evidence of SARS-CoV-2 exposure in rodents. Large-scale surveys have consistently failed to detect viral RNA by RT-PCR, while sporadic serological signals, such as neutralizing antibodies detected in a small number of rats and mice, have frequently lacked molecular confirmation or produced indeterminate results [14,15]. These patterns have been attributed to diagnostic challenges, including assay variability, low viral loads, and potential cross-reactivity with endemic rodent coronaviruses, as documented in metagenomic and serological studies in urban rodents [16]. Taken together, these findings highlight both the rarity of confirmed SARS-CoV-2 infections in rodents and the importance of region-specific wildlife surveillance to better understand reverse zoonotic dynamics.

Spillover events have been strongly associated with land-use change, including urbanization, agricultural intensification, deforestation, fragmentation, and habitat loss [17]. Such transformations alter biodiversity structure and species interactions while increasing contact rates between wildlife and humans, thereby facilitating pathogen transmission across species, in both zoonotic and reverse zoonotic directions [18]. The effects of habitat modification on pathogen transmission have been widely studied, with high rates of habitat conversion generally associated with increased transmission risk among species occupying conserved–modified landscape mosaics [19,20]. Tropical regions, harbor which high global biodiversity, are currently experiencing rapid habitat transformation. These changes intensify human–wildlife interactions and are recognized as key drivers of pathogen spillover and the emergence of zoonotic diseases [21].

The Yucatan Peninsula harbors high biological diversity and has experienced extensive deforestation in recent decades [22,23]. Between 2020 and 2022, 305,667 confirmed cases of COVID-19 were reported in the region [24]. The COVID-19 pandemic therefore provides an opportunity to explore pathogen dynamics across species, including the potential for reverse zoonotic transmission in wildlife. In this context, we conducted an exploratory field survey to detect evidence of SARS-CoV-2 exposure in rodent communities across multiple rural localities in the Yucatan Peninsula, providing an initial assessment of possible reverse zoonotic dynamics in this region.

## 2. Materials and Methods

### 2.1. Study Area and Sampling Design

Sampling was conducted during 2021 and 2022 in eight rural localities across the Yucatan Peninsula (Table 1). These communities are primarily characterized by agricultural, livestock, and forest resource use activities, which often involve close interactions among humans, domestic animals, and wildlife.

Rodents were captured using Sherman traps (8 × 8 × 23 cm; H.B. Sherman traps, Tallahassee, FL, USA). Traps were arranged in four quadrants, each consisting of a 7 × 7 grid with 10 m spacing between traps, they were kept active for 3 consecutive nights at each site. Captured rodents were taxonomically identified, morphometric measurements, age, sex, and reproductive status were recorded. Blood samples (~0.1 mL) were obtained by puncture of the retro-orbital sinus using capillary tubes and preserved on filter paper strips (Cole-Parmer, Vernon Hills, IL, USA). Rectal samples were collected using rayon swabs (Puritan Medical Products, Guilford, ME, USA), preserved in RNAlater (Qiagen, Hilden, Germany), and stored at −80 °C for subsequent molecular analyses. After sample collection, individuals were marked with a numbered metal tag and released at the site of capture.

All capture, handling, and sampling procedures were conducted in accordance with the Guidelines of the American Society of Mammalogists for the use of wild mammals in research and education [25], under license FAUT-0250 issued by the Secretary of Environment and Natural Resources, Mexico (Secretaría de Medio Ambiente y Recursos Naturales, México) and approved by the Institutional Subcommittee for the Care and Use of Experimental Animals of the Faculty of Veterinary Medicine and Zootechnics, National Autonomous University of Mexico (SICUAE.DC-2022/2-2).

### 2.2. Indirect ELISA

As antigen, we used five peptides in MAP8 (multiantigenic peptide) format derived from the receptor-binding domain (RBD) of the SARS-CoV-2 spike protein, together with recombinant RBD protein expressed in the *Rosetta* strain of *Escherichia coli*, as previously described by Cortés-Sarabia et al. [26,27]. The ELISA assay was originally developed and validated for the detection of anti-SARS-CoV-2 antibodies in human sera, where it demonstrated a sensitivity of 92% and specificity of 96% [27]. Although the assay has not been formally validated for wild rodent species, it was adapted for use in rodent sera given its capacity to detect antibodies across mammalian species through Protein A/G binding [28].

Microtiter plates (Sigma-Aldrich, St. Louis, MO, USA) were coated with 100 µL/well of both antigens to a final concentration of 0.1 µg/mL in coating buffer (50 mM Na_2_CO_3_/NaHCO_3_, pH 9.6). Plates were incubated for 1 h at 37 °C and subsequently blocked for 40 min at 37 °C with 200 µL/well of 5% skimmed milk diluted in phosphate-buffered saline (PBS)-Tween 20 (0.05%). Rodent serum samples were tested in duplicate at a dilution of 1:25 (100 µL/well) and incubated for 1 h at 37 °C. Bound antibodies were detected using horseradish peroxidase (HRP)-conjugated Protein A/G 100 µL/well (Thermo Scientific Pierce, Rockford, IL, USA; catalog no. 32490), incubated for 30 min at 37 °C. Protein A/G was selected due to its ability to bind the Fc region of IgG from multiple mammalian species, including rodents. After each step, plates were washed three times with 200 µL/well of PBS-Tween 20 (0.05%) for 5 min. The enzymatic reaction was developed using o-phenylenediamine dihydrochloride (Sigma-Aldrich) and stopped with 2 N H_2_SO_4_. Optical density (OD) was measured at 492 nm using a microplate reader (Epoch, BioTek Instruments, Winooski, VT, USA). Positive controls included sera from SARS-CoV-2–infected and vaccinated humans, as well as sera from New Zealand rabbits immunized with RBD-MAP8 peptides. Negative controls consisted of sera from BALB/c mice. Adjusted OD values were calculated by subtracting the mean OD of negative controls from each sample. The positivity cut-off was defined as the mean OD of negative controls plus three standard deviations. Samples with adjusted OD values greater than 0.254 were considered seroreactive.

Seroreactivity frequency (*SRF*) was calculated as the proportion of seroreactive individuals (*N_ir_*) relative to the total number of individuals analyzed (*N_i_*). To reduce the influence of variation in the number of individuals analyzed per species and locality, seroreactivity frequency was weighted (*SRF_w_*) according to the number of individuals analyzed (*N*_i_) log10 transformed using the following formula, this approach reduces the overrepresentation of species and localities with large sample sizes [29,30,31].

$$SRFw\;=\;log10\left(Ni\right)\;\times \;SRF$$

### 2.3. Molecular Detection

Viral RNA was extracted from rectal swabs using the QIAamp Viral RNA Mini Kit (QIAGEN, Hilden, Germany), following the manufacturer’s protocol. Detection of coronavirus (CoV) RNA was performed using a pan-CoV nested reverse transcription polymerase chain reaction (RT-PCR) assay targeting a 440 bp region of the RNA-dependent RNA polymerase (RdRP) gene, as described by Chu et al. [32]. Primer sequences were used as originally described by Chu et al. [32], and reaction components followed the published protocol. cDNA synthesis and the first-round PCR were conducted using the OneStep SuperScript III kit (Invitrogen, Carlsbad, CA, USA). The second-round PCR reaction was performed with HotStarTaq Plus DNA Polymerase (QIAGEN, Hilden, Germany). Cycling conditions followed the published protocol, except that the annealing temperature in the second round was adjusted to 54 °C. Each PCR run included a positive control consisting of total RNA of a *Porcine epidemic diarrhea virus* (PEDV), strain MT490316.1 (GenBank), used as positive control and a negative control to monitor contamination (complete reaction without a template). PCR products were visualized on a 2% agarose gel stained with SYBR Safe DNA Gel Stain (Invitrogen). Samples were considered positive when a single band corresponding to the expected size (440 bp) was observed. Any sample showing amplification in the pan-CoV assay was subsequently analyzed using a SARS-CoV-2 specific RT-PCR assay described by Corman et al. [33], targeting the RdRP gene and E gene. Reaction conditions followed the original protocol, and total RNA of a SARS-CoV-2 positive human sample, kindly provided by the laboratory of Sofía L. Alcaraz-Estrada, was included as a positive control, together with a negative control.

## 3. Results

**Figure 1:**
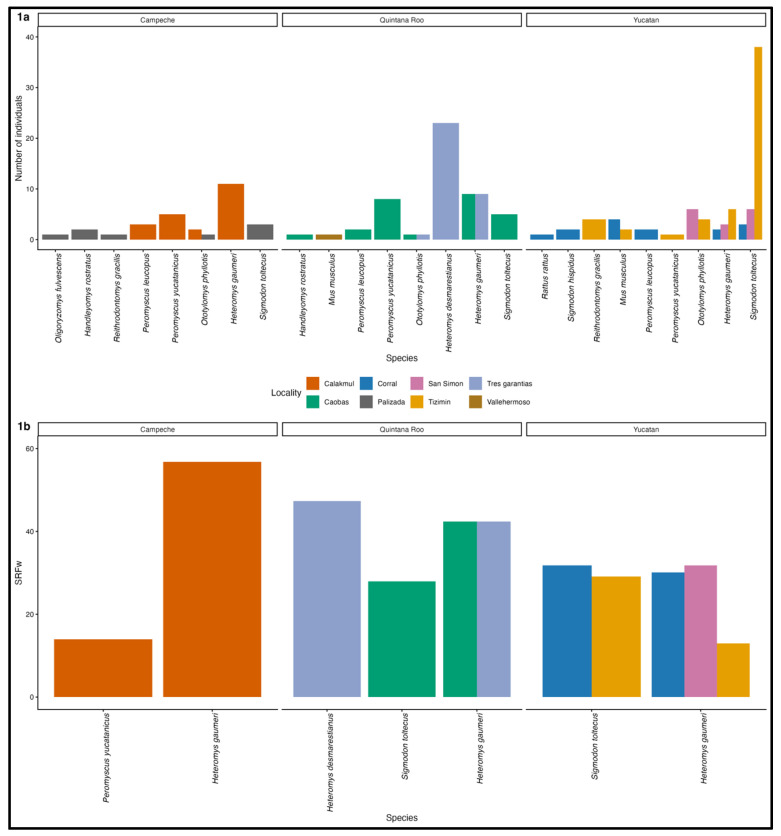
(**a**) Rodent species recorded. (**b**) Weighted seroreactivity frequency (SRF_w_) by species and State. *Re. gracilis*, represented by a single sampled individual that tested seroreactive, was not plotted because its weighted seroreactivity frequency value was zero.

A total of 173 rodents were recorded across 12 species belonging to three families (*Muridae*, *Cricetidae*, and *Heteromydae*) distributed in eight localities. The state with the highest rodent capture was Yucatan (84), followed by Quintana Roo (60), and finally, Campeche recorded the lowest number of rodents (29). Tizimin and Tres Garantias recorded the highest number of individuals (55 and 33, respectively), while Palizada and Vallehermoso recorded the lowest (8 and 1, respectively). The most represented species were *Sigmodon toltecus* and *Heteromys gaumeri*, while the least represented species were *Oligoryzomys fulvescens* and *Rattus rattus* (Figure 1a).

Of all sera analyzed, 40 were seroreactive (23.12%) for SARS-CoV-2, distributed across five species with at least one seroreactive individual, *Heteromys desmarestianus*, *H. gaumeri*, *Peromyscus yucatanicus*, *Reithrodontomys gracilis* and *S. toltecus* (Figure 1b). Seroreactivity frequency was highest in Quintana Roo (SRFw 53.34), lowest in Yucatán (SRF_w_ 31.49), and intermediate in Campeche (SRF_w_ 42.41). At the locality level, the highest seroreactivity frequency was observed in Tres Garantias and the lowest in Palizada (Table 2).

A total of 162 viable rectal swab samples were obtained for molecular analysis. Of these, only one individual, from the species *H. gaumeri* collected in a conserved habitat in Campeche, tested positive by RT-PCR using the pan-CoV protocol, but negative to the SARS-CoV-2 specific protocol.

## 4. Discussion

Our study detected SARS-CoV-2 reactive antibodies in rodents from three families—Cricetidae, Heteromyidae, and Muridae—across rural localities of the Yucatan Peninsula. Although overall seroreactivity was heterogeneous across species, antibody detection was notable when contrasted with the generally low serological signals reported in wildlife surveillance studies conducted to date. Indeed, SARS-CoV-2 seroprevalence reported in wild rodent surveillance studies worldwide has been consistently low, generally ranging from 0% to <1%. Large-scale surveys in Europe and South America reported either no seropositive individuals or sporadic detections below 1%, frequently in the absence of molecular confirmation [34,35]. A European multi-country study reported a single *Apodemus sylvaticus* individual testing positive by immunofluorescence; however, this result was not confirmed by seroneutralization or microsphere immunoassay [34]. To our knowledge, this study represents the first report of SARS-CoV-2 exposure in rodent communities from non-urbanized habitats in Mexico. Seroreactivity was particularly concentrated in *H. gaumeri*, *H. desmarestianus*, and *P. yucatanicus*, an endemic species of the Yucatan Peninsula, suggesting non-random exposure patterns rather than uniform background reactivity.

None of the murid rodents sampled in our study tested seroreactive. Other studies analyzing murid rodents in urban and rural habitats have reported variable results. It is important to note that our study did not include urban habitats and that murids were the least represented family. Consequently, future surveillance efforts should prioritize monitoring SARS-CoV-2 in urban rodent populations across the Yucatan Peninsula as an important component of comprehensive wildlife disease surveillance. This is particularly relevant given that in Mexico City, SARS-CoV-2 has been detected in *Mus musculus* and *Rattus norvegicus* by RT-qPCR, with prevalences of 12.1% and 5.8%, respectively [36]. Similarly, a study in Belgium found three urban *R. norvegicus* individuals seropositive by immunoassay, although viral neutralization and RT-qPCR tests were negative [37].

Cricetid rodents include species that can act as reservoirs of zoonotic viruses, such as *Peromyscus* and *Sigmodon*. The species from these genera are considered tolerant to habitat disturbances, occurring in both conserved and peridomestic areas. Experimental studies show that *Peromyscus maniculatus* is susceptible to SARS-CoV-2, supporting viral replication, shedding, and transmission, though clinical signs are generally absent [9,38,39]. In contrast, some *P. californicus* individuals developed disease [39]. In our study, seroreactivity was detected in *P. yucatanicus*, an endemic species of the Yucatan Peninsula. Given the documented susceptibility of *Peromyscus* species to SARS-CoV-2, *P. yucatanicus* may represent a susceptible host capable of mounting an antibody response following exposure. Whether this species could contribute to sustained viral circulation in natural settings remains unknown and warrants targeted longitudinal surveillance.

Seroreactivity was also observed in *S. toltecus* (11/62) and *S. hispidus* (1/3). Members of this genus have been used as models for SARS-CoV-2 infection under laboratory conditions, where *S. fulviventer* exhibited multiorgan and multisystemic effects and developed neutralizing antibodies [40]. These findings suggest that *Sigmodon* species may be permissive hosts for SARS-CoV-2 exposure rather than reservoirs sustaining long-term transmission cycles under natural conditions. Nevertheless, continued monitoring is crucial, as infection could influence population dynamics and ecosystem function. Notably, seroreactive species in this study display behavioral flexibility, high ecological plasticity, and tolerance to disturbance, whereas habitat specialists tested negative.

*H. gaumeri*, *H. desmarestianus*, *Re. gracilis*, and *S. toltecus* are well adapted to open habitats and frequently occur in cultivated areas. Although *P. yucatanicus* is less tolerant of disturbance and prefers forested or closed habitats, it is often present in peridomestic areas of rural communities [41,42,43]. The co-occurrence of these species with humans, coupled with frequent interactions in rural settlements, may increase opportunities for reverse zoonotic transmission and incidental exposure. Such interface dynamics could favor sporadic spillback events across heterogeneous landscapes. Notably, the Yucatan Peninsula experienced periods of high SARS-CoV-2 incidence during the pandemic [44], which may have increased the probability of human-to-wildlife exposure events in this region. While a direct link cannot be established, the regional epidemiological context provides a plausible background for the seroreactivity detected in rodents.

The expansion of human activities—including population growth, habitat intrusion, and environmental pollution—contributes to biodiversity loss and facilitates pathogen transmission at the human–wildlife interface. These processes may increase infection risk or favor pathogen adaptation to certain species, potentially leading to the establishment of new reservoirs and amplifying zoonotic disease emergence [45,46]. Goldberg et al. detected SARS-CoV-2 antibodies in wildlife, including *P. maniculatus* and *P. leucopus*, and reported a positive correlation between urbanization and seroprevalence, with the highest antibody detections occurring in one of the least urbanized sites [47]. The localities included in our study, although partially conserved, include human settlements and domestic animals such as cats, which are susceptible to SARS-CoV-2 and may facilitate viral transmission to co-occurring wildlife.

Although SARS-CoV-2, SARS-CoV, and MERS-CoV generally do not cause severe disease in rodents, experimental studies indicate that wild-type strains replicate transiently and often at low levels in conventional rodent models, with viral detection typically restricted to a narrow temporal window following infection [48,49]. This transient replication pattern may contribute to the frequent absence of molecular detection in field-based studies despite serological evidence of prior exposure. Consistent with these experimental observations, the lack of SARS-CoV-2–specific RNA detection in rectal swabs in our study does not preclude previous infection. Viral shedding in rodents appears to be short-lived and tissue-specific, and gastrointestinal shedding may be limited or inconsistent. Therefore, rectal or fecal sampling may have a reduced probability of detecting active infection outside the acute phase. Under these conditions, serological assays provide a more reliable indicator of past exposure than RT-PCR–based approaches. Such discordant serological and molecular patterns are consistent with interpretations of prior spillback events rather than evidence of sustained viral circulation in wildlife populations [13,16,35].

In our study, only one rodent tested positive using a pan-coronavirus protocol, while all were negative for SARS-CoV-2–specific assays, possibly reflecting limited gastrointestinal shedding, as observed in *P. maniculatus* [9]. Other studies have also reported an absence of active infection and very low seroreactivity frequency in rodents sampled across urban, rural, and natural habitats, suggesting localized exposure influenced by environmental or anthropogenic factors [35]. Comparable serological patterns have been described in rodents from Sarawak, Malaysia, where neutralizing antibodies against SARS-CoV-2 were detected in *Sundamys muelleri* and *R. rattus* using archived plasma samples collected between 2018 and 2022, in the absence of evidence for sustained rodent-to-rodent transmission [13]. Although ecological contexts differ, the concurrence of serological reactivity without molecular confirmation in geographically distant rodent communities suggests that wildlife exposure may occur as sporadic, localized spillback events. Taken together, these observations are more consistent with incidental reverse zoonotic exposure than with the establishment of stable sylvatic transmission cycles. However, while serological evidence provides valuable insights into prior exposure, it does not allow determination of viral origin or transmission pathways. Definitive assessment of SARS-CoV-2 sources, host adaptation, and potential transmission dynamics would require genomic characterization of viral sequences, which remains challenging in wildlife studies due to logistical and resource constraints.

Given these limitations, serological assays remain essential for detecting past exposure to SARS-CoV-2 in wildlife. A potential limitation of serological surveillance is cross-reactivity with endemic murine coronaviruses. However, available evidence suggests that cross-reactivity in assays targeting the spike protein—particularly the receptor-binding domain (RBD)—is limited, as neutralizing and binding antibodies against SARS-CoV-2 predominantly recognize virus-specific epitopes within the S1 subunit [50,51].

Additionally, the use of a chimeric Protein A/G conjugate provides an expanded IgG-binding spectrum relative to Protein A alone. Experimental studies have demonstrated that Protein A exhibits subclass-dependent binding in rodents, with limited affinity for certain murine IgG subclasses and poor reactivity with rat IgG, whereas Protein G displays broader binding across murine and rat IgG isotypes [52]. The combined Protein A/G format has therefore been widely applied in multispecies serological assays to overcome species-specific limitations [28,53]. Its utility has also been demonstrated in wildlife and rodent-based viral surveillance studies, including hantavirus and experimental infection models [54,55]. Nevertheless, interspecific variation in IgG structure and subclass distribution may still influence relative binding efficiency and assay sensitivity. Consequently, differences in seroreactivity frequency observed among rodent species in our study should be interpreted with caution, given the potential variability in antibody-binding affinity. However, the heterogeneous and non-random distribution of seroreactivity across species and localities argues against a generalized methodological artifact.

Taken together, our findings indicate serological evidence of SARS-CoV-2 exposure in rodent communities inhabiting rural landscapes of the Yucatan Peninsula, in the absence of molecular confirmation of active infection. The non-random distribution of seroreactivity across species and localities suggests discrete exposure events rather than generalized background reactivity. Within the broader context of increasing human–wildlife interface dynamics, such patterns underscore the importance of longitudinal, multispecies surveillance frameworks. Integrating ecological, epidemiological, and molecular approaches under a One Health perspective will be essential to better characterize the frequency, drivers, and consequences of reverse zoonotic events in heterogeneous landscapes. Continued monitoring will help clarify whether such exposures remain sporadic or represent early signals of more complex host–pathogen interactions in wildlife communities.

## 5. Conclusions

This study documents serological evidence of exposure to SARS-CoV-2 exposure in rodent communities from rural areas of Mexico. Observed exposure patterns were heterogeneous across species and localities, potentially reflecting the influence of human activities, species adaptability to disturbed habitats, and frequent human–rodent interactions. While direct human-to-rodent transmission is plausible, indirect pathways involving intermediary hosts may also contribute to these exposure patterns.

Although molecular confirmation of active infection was not detected, the serological profiles observed are consistent with sporadic spillback events rather than sustained transmission in wildlife populations. These findings highlight the importance of continued wildlife surveillance integrating serological and molecular approaches to better characterize viral circulation and clarify the ecological role of rodents as exposed hosts within broader transmission networks. Future studies should examine how SARS-CoV-2 exposure is established and maintained across time and space within socio-ecological systems, recognizing humans as integral components and reverse zoonosis as an example of the interconnectedness of human, animal, and environmental health.

## Figures and Tables

**Table 1:** Localities in the Yucatan Peninsula.

State	Municipality	COVID Cases ^†^	Locality	Latitude	Longitude
Campeche	Calakmul	706	Conhuas	18.48145	−89.89042
	Palizada	366	Palizada	18.09279	−91.9305
Quintana Roo	Othon P. Blanco	17,541	Tres Garantias	18.20881	−89.05823
	Othon P. Blanco		Caobas	18.39099	−88.99862
	Bacalar	815	Vallehermoso	19.15369	−88.53938
Yucatan	Santa Elena	61	San Simon	20.21113	−89.78382
	Tzucacab	213	Corral	19.81034	−88.99614
	Tizimin	3023	Tizimin	21.15324	−87.91073

^†^ Reported cases from 2020 to October 2022 per municipality.

**Table 2:** Seroreactivity frequency per locality.

State	Locality	AI	SRI	SRF (%)	SRFw
Campeche	Calakmul	21	7	33.33	44.07
	Palizada	8	1	16.67	12.97
Quintana Roo	Tres Garantias	33	12	36.36	55.22
	Caobas	26	6	23.08	32.65
	Vallehermoso	1	0	0	0
Yucatan	San Simon	15	2	13.33	15.68
	Corral	14	4	25	30.10
	Tizimin	55	8	14.55	25.31

Analyzed individuals (AI), Seroreactive individuals (SRI), Seroreactivity frequency (SRF), Weighted Seroreactivity frequency (SRFw).

## Data Availability

The original contributions presented in this study are included in the article. Further inquiries can be directed to the corresponding author.
